# Circadian Regulation of Mitochondrial Uncoupling and Lifespan

**DOI:** 10.1093/geroni/igaa057.2648

**Published:** 2020-12-16

**Authors:** Matthew Ulgherait

**Affiliations:** CUMC, New York, New York, United States

## Abstract

Because old age is associated with defects in circadian rhythm, loss of circadian regulation is thought to be pathogenic and contribute to mortality. We show instead that loss of specific circadian clock components Period (Per) and Timeless (Tim) in male Drosophila significantly extends lifespan. This lifespan extension is not mediated by canonical diet-restriction longevity pathways, but is due to altered cellular respiration via increased mitochondrial uncoupling. Lifespan extension of per mutants depends on mitochondrial uncoupling in the intestine. Moreover, up-regulated uncoupling protein UCP4C in intestinal stem cells and enteroblasts is sufficient to extend lifespan and preserve proliferative homeostasis in the gut with age. Consistent with inducing a metabolic state that prevents over-proliferation, mitochondrial uncoupling drugs also extend lifespan and inhibit intestinal stem cell overproliferation due to aging or even tumorigenesis. These results demonstrate that circadian-regulated intestinal mitochondrial uncoupling controls longevity in Drosophila and suggest a new potential anti-aging therapeutic target.

